# Potential involvement of peroxisome proliferator-activated receptors in the inhibition of mammary lipid synthesis during diet-induced milk fat depression

**DOI:** 10.3168/jds.2024-25575

**Published:** 2024-11-08

**Authors:** A. Haile, D. E. Oliveira, Y. R. Boisclair, D. E. Bauman, K. J. Harvatine

**Affiliations:** 1 Department of Animal Science, Penn State University, University Park, PA 16802; 2 Department of Animal Production, Santa Catarina State University, Lages, Santa Catarina, 88520-000, Brazil; 3 Department of Animal Science, Cornell University, Ithaca, NY 14850

**Keywords:** conjugated linoleic acid, PPAR-γ, biohydrogenation

## Abstract

The objective of this study was to evaluate the possible role of the peroxisome proliferator-activated receptors (PPAR: PPAR-α, PPAR-β/δ, and PPAR-γ) in diet and CLA-induced milk fat depression (MFD) in dairy cows. We hypothesized that the expression of PPAR, which regulate lipid metabolism and bind to PUFA, could be modulated by biohydrogenation intermediates that induce MFD, thereby interfering with milk fat synthesis. First, tissue profiling revealed that PPAR-α and PPAR-β/δ had low expression in mammary tissue compared with the liver. A comparison of lactating and nonlactating tissue from the same cows showed that expression of all 3 PPAR isoforms did increase during lactation. Mammary expression of the *PPAR* family during MFD was then observed in 9 mid-lactation cows in a 3 × 3 Latin square design with MFD induced by a 3-d intravenous infusion of *trans*-10,*cis*-12 CLA or feeding a high-oil and low-forage diet. The expression of all 3 PPAR isoforms remained largely unaltered during CLA and diet-induced MFD, except for an increase in PPAR-α target genes *CPT1A* and *ACADVL* that are involved in β-oxidation. The interaction of PPAR-γ chemical agonist troglitazone and antagonist T0070907 and CLA was then investigated in bovine mammary epithelial cells. The activation and inhibition of PPAR-γ did not overcome *trans*-10,*cis*-12 CLA inhibition of lipogenesis despite the agonist stimulating PPAR-γ expression. Furthermore, PPAR-γ activation did not modify the expression of lipogenic genes. Overall, the results fail to support a functional role of the PPAR family in the inhibition of lipogenesis during MFD in dairy cows.

## INTRODUCTION

Milk fat depression (**MFD**) is a physiological alteration that occurs when ruminants consume diets high in fermentable carbohydrates and unsaturated fatty acids (UFA). This results in a substantial reduction in milk fat yield, primarily caused by specific bioactive intermediates of fatty acid (**FA**) metabolism that arise from altered ruminal biohydrogenation. Among these, *trans-*10,*cis-*12 CLA is the most extensively studied due to its impact on MFD and commercial availability (Harvatine et al., 2009; Bauman et al., 2011).

The mammary gland’s capacity for lipogenesis is notably diminished during MFD and is accompanied by a coordinated downregulation of key lipogenic signaling factors, such as sterol response element binding protein 1 (*SREBF1*) and thyroid hormone responsive thyroid hormone responsive (*THRSP*), and crucial lipogenic enzymes, such as acetyl-CoA carboxylase (*ACACA*), fatty acid synthase (*FASN*), and lipoprotein lipase (*LPL*; Harvatine et al., 2009). The observed downregulation suggests a complex interplay between diet and gene expression, yet the precise mechanisms responsible remain poorly understood.

The peroxisome proliferator-activated receptors (**PPAR**) are members of the nuclear hormone receptor family, have FA binding pockets, and are regulators of lipid metabolism in other model systems (Janani and Kumari, 2015; Bougarne et al., 2018; Szántó et al., 2021). They have also been proposed as a regulator of milk fat synthesis and to play a causative role in MFD (Bionaz et al., 2013). There are 3 PPAR isoforms, PPAR-α, PPAR-β/δ, and PPAR-γ, and each forms a heterodimer with activated retinoid X receptors (**RXR**) after ligand activation and binds to peroxisome proliferator regulatory elements (**PPRE**) in the promoters of responsive genes. PPAR-α is primarily expressed in tissues with high rates of FA oxidation, such as the liver, muscle, and intestine (Montaigne et al., 2021). PPAR-β/δ exhibits ubiquitous expression across tissues (Kadayat et al., 2020), whereas PPAR-γ is mainly found in adipose tissue and macrophages. Each PPAR has distinct roles in lipid metabolism and energy homeostasis (Janani and Kumari, 2015).

Intriguingly, intracellular free FA, including specific isomers of CLA, have been reported to be ligands for the PPAR (Yuan et al., 2015; Murru et al., 2021). Specifically, studies using transactivation assays have demonstrated that *trans*-10,*cis*-12 CLA, *cis-*9,*trans-*11 CLA, and linoleic acid all similarly activate PPAR-α, PPAR-β/δ, and PPAR-γ, yet only that *trans*-10,*cis*-12 CLA caused MFD (Belury et al., 2002; Yu et al., 2002; Kim et al., 2011; Benjamin et al., 2013). Additionally, Ma et al., (2014) observed that *cis-*9,*trans-*11 CLA, *trans-*10,*cis-*12 CLA, and *cis-*9,*cis-*12 linoleic acid similarly increased activity of a PPRE luciferase promoter construct in bovine mammary epithelial cells. The activation of PPAR-α would be expected to result in increased lipid oxidation (Bougarne et al., 2018). It is important to note that activation of PPAR-γ would be expected to increase fat synthesis based on the response in adipose tissue (Janani and Ranjitha Kumari, 2015; Blanchard et al., 2016), and activation by FA and CLA isomers not associated with MFD is puzzling. The role of PPAR isoforms in regulating mammary lipid metabolism has been investigated in mammary cell culture and other animal models. Although a functional role has not been demonstrated, PPAR-γ remains a common proposed mechanism (Osorio et al., 2016).

Recognizing the pivotal role of the PPAR family in lipid metabolism, this study aimed to evaluate the potential role of PPAR isoforms in MFD in the cow through multiple levels of investigation. We first conducted tissue profiling to determine the expression of the PPAR isoforms in the mammary gland relative to other lipogenic tissues. Next, we hypothesized that if they were an important regulator of mammary lipid metabolism, they would be more highly expressed in lactating than nonlactating mammary tissue. We then determined the effect of diet- and CLA-induced MFD on mammary expression of the 3 PPAR isoforms and PPAR-responsive genes using samples available from a previously conducted experiment. Lastly, because PPAR-γ is the most closely linked with lipid synthesis in other tissues, the interaction of PPAR-γ activation and inhibition and CLA was investigated in mammary cell culture, with the hypothesis that if CLA functioned through PPAR-γ activation or inhibition, its impact would be modified by PPAR-γ agonist or antagonist.

## MATERIALS AND METHODS

### Samples from Previous Animal Experiments

Ethical approval for experimental procedures was obtained from the Cornell University Institutional Animal Care and Use Committee. Tissue samples for spatial expression analysis were previously collected from lactating Holstein cows following humane slaughter via exsanguination after stunning with a captive bolt. The tissues available included subcutaneous adipose tissue, liver, skeletal muscle, heart, uterine, lung, and brain. Additionally, mammary tissue samples were collected by needle biopsy from 7 cows both before lactation (~30 d prepartum) and during established lactation (240 ± 85 DIM; mean ± SD) as previously described by Harvatine and Bauman (2006).

To study the effects of diet-induced and CLA-induced MFD on mammary gene expression, we used mammary tissue samples from a previous experiment that focused on the effect of MFD on the expression of lipogenic enzymes, SREBF1, and thyroid hormone responsive spot 14 (Harvatine and Bauman, 2006). The experiment used 9 mid-lactation cows (193 ± 32 DIM; mean ± SD) in a 3 × 3 Latin square design with 14-d experimental periods. Briefly, the treatments included a control group fed a 64.7% forage diet with no added fat, a group fed the control diet while receiving an infusion of 10 g/d of *trans*-10,*cis*-12 CLA for 3 d, and a group fed a low-forage and high-oil diet (**LF-HO**; 45.9% forage, 3.0% soybean oil, and 1.5% fish oil) to induce MFD. The CLA infusion was prepared using emulsified methyl ester stock with 88.3% total CLA (98% *trans-*10,*cis-*12 CLA isomer). As previously reported, milk fat concentration and yield were reduced by 23% and 24%, respectively, in the CLA-treated group, and by 31% and 38% in the LF-HO diet group (Harvatine and Bauman, 2006). Additionally, both de novo and preformed FA were reduced, with de novo FA exhibiting a greater decrease. Mammary needle biopsies were performed 1 to 3 h after milking, tissues were snap-frozen in liquid nitrogen, stored at −80°C, and RNA extracted as described below.

### Cell Culture

Bovine mammary epithelial cells (MAC-T) were cultured as described by Harvatine et al. (2014). Briefly, cells were grown to 80% to 90% confluence in basal medium (Dulbecco’s modified Eagle’s medium [DMEM] supplemented with 5 mM sodium acetate, 5 mM Glutamax [Invitrogen, Carlsbad, CA], 20 IU/mL of penicillin and streptomycin [Invitrogen], 10% fetal calf serum [Gemini Bio-products, West Sacramento, CA], and 5 μg/mL bovine insulin [Sigma, St. Louis, MO]). Cells were incubated in basal media without serum and insulin for 24 h before treatments applied. Treatments included 5 μM PPAR-γ agonist troglitazone (TG; Calbiochem, San Diego, CA), 5 μM PPAR-γ antagonist T0070907 (T07; Calbiochem, San Diego, CA), 10 μM cis-9 retinoic acid (9cRA; Sigma, St. Louis, MO), and 75 μM trans-10,cis-12 CLA, either individually or in combination. The CLA treatment used a free FA stock comprising 95.3% trans-10,cis-12 CLA and 2.2% linoleic acid (Larodan AB, Malmö, Sweden), complexed to BSA via a method using potassium salt formation (Keating et al., 2008). Lipogenesis was assessed by measuring 14C acetate incorporation into FA as described by Harvatine et al. (2014). Experiments included 3 wells of each treatment, and experiments were replicated on 2 occasions.

### RNA Isolation and Real-Time PCR

Total RNA was isolated and gene expression measured by real-time PCR as described by Harvatine and Bauman (2006), with minor adaptations. Briefly, total RNA was extracted using the RNeasy Lipid Kit with on-column DNase treatment (Qiagen). Reverse transcription was conducted using SuperScript III and random primers (Invitrogen). The PCR reactions included ABI Power SYBR with ROX (ABI) and 400 nm of gene-specific forward and reverse primers. Expression levels were determined by comparing sample expression to a dilution curve of pooled cDNA (Applied Biosystems, 2008).

### Statistical Analysis

Data analysis was performed using the fit model procedure of JMP Pro (SAS Institute Inc., Cary, NC). The model for tissue expression included the tissue type and the geometric mean of 3 housekeeping genes (*RNA18SN1*, *ACBT*, and *B2M*). For comparison of lactating and nonlactating mammary tissues, the model included the random effect of cow and fixed effect of lactation state and the geometric mean of 3 housekeeping genes (*ACTB*, *B2M*, and *RPS9*). The model for the in vivo Latin square experiment investigating MFD included the random effect of cow and period and the fixed effect of treatment and the geometric mean of the housekeeping genes. Pre-planned contracts compared the control to CLA treatment and control to the LF-HO diet. The model to analyze cell culture experiments included the random effect of the experiment replicate and the fixed effect of treatment, and means were separated using a protected LSD. In all experiments, data were log-transformed when residuals were not uniformly distributed and back-transformed data are reported.

## RESULTS

### Spatial Expression of PPAR Isoforms

**Figure 1.**
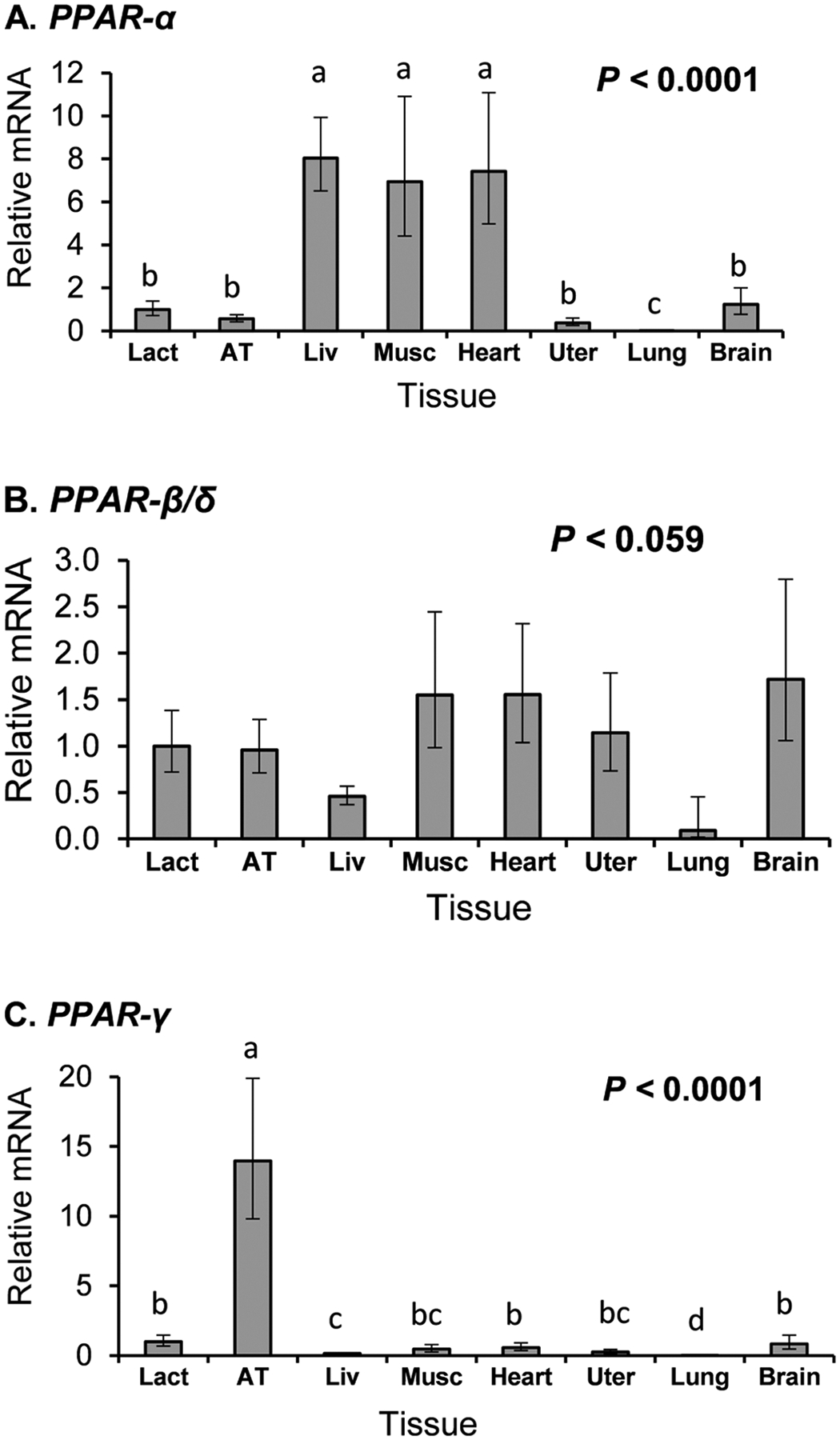
Tissue profile of the peroxisome proliferator-activated receptors in the cow. Tissue expression (*PPAR-α*, panel A; *PPAR-β/δ*, panel B; and *PPAR-γ*, panel C) in the mid- to late-lactation cows (n = 6 for subcutaneous adipose tissue [AT], liver [Liv], and lactating mammary gland [Lact], and n = 3 for uterus [Uter], lung, brain, skeletal muscle [Musc], and heart). Least squares means and SEM are shown. Means that do not share a letter differ (*P* < 0.05).

Comparative analysis of tissues obtained from lactating cows revealed distinct patterns of PPAR isoform expression (Figure 1). *PPAR*-α was predominantly expressed in the liver, muscle, and heart, each being over 5-fold higher than lactating mammary tissue. *PPAR*-β/δ expression was not different between tissues (*P* = 0.06) and was numerically similar in lactating tissue as adipose, muscle, heart, uterine, and brain. *PPAR*-γ was mainly expressed in adipose tissue, where its expression was over 12-fold higher than lactating mammary tissue.

**Figure 2.**
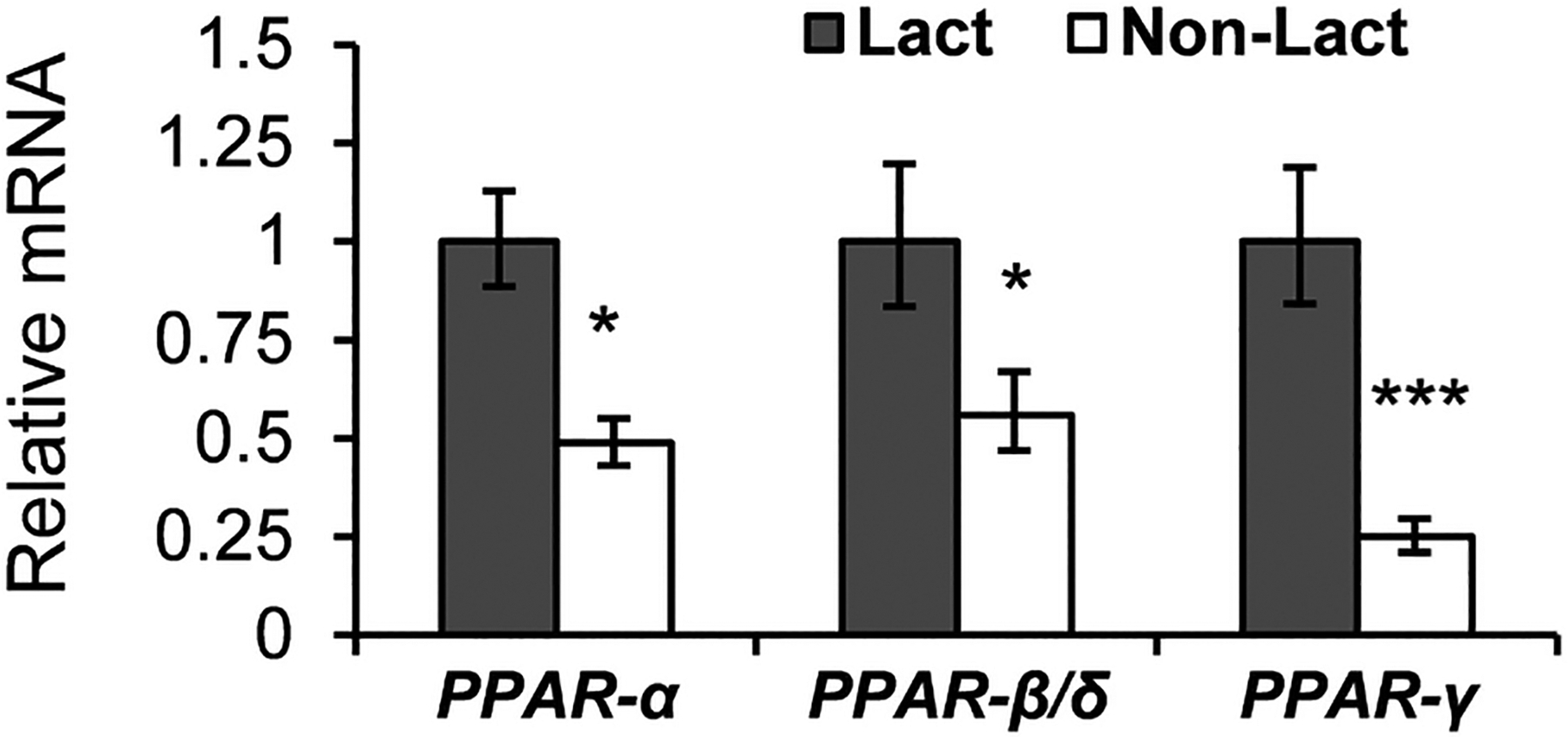
Expression in lactating and nonlactating tissue (n = 7 cows sampled in both states). Values represent LSM ± SEM scaled relative to lactating tissue (set to 1). Significant differences between lactating and nonlactating tissue is indicated (*P < 0.05; ***P < 0.001).

All 3 PPAR isoforms were more highly expressed in lactating mammary tissue compared with nonlactating tissue from the same cows (Figure 2). Specifically, mammary expression of *PPAR-*γ was 2-fold higher in lactating than nonlactating mammary tissue (*P* < 0.01) and *PPAR*-α and *PPAR*-β/δ were ~1-fold higher (*P* < 0.05).

### Effect of MFD on Mammary Expression of PPAR Isoforms and PPAR Target Genes

**Figure 3.**
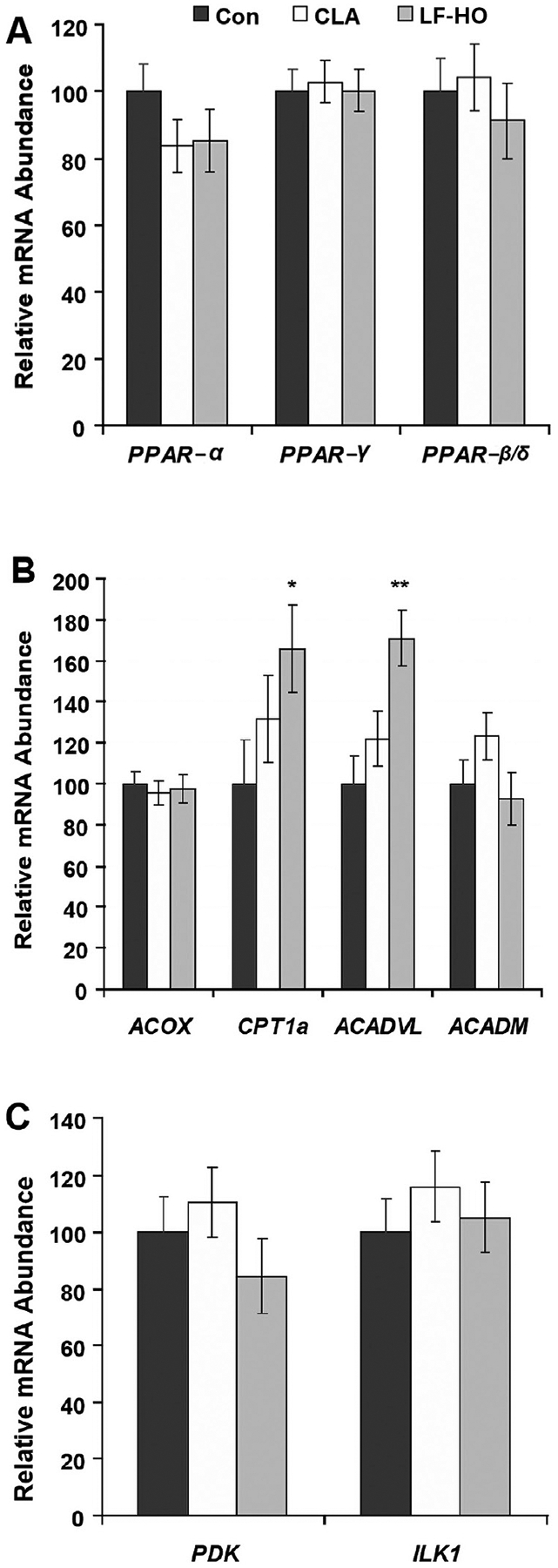
Effects of milk fat depression induced by *trans-*10,*cis-*12 CLA or a low-forage, high-oil diet (LF-HO) on mammary expression of peroxisome proliferator-activated receptors (*PPAR*) and PPAR-responsive genes in dairy cows. Milk fat yield was reduced 24% by CLA and 38% by the LF-HO diet compared to control (Con; Harvatine and Bauman, 2006). (A) Expression of *PPAR-*α, *PPAR-*β/δ, and *PPAR-*γ. (B) PPARα responsive fatty acid oxidation enzymes including acyl-coA oxidase (*ACOX*), carnitine palmitoyltransferase 1 (*CPT1a*), acyl-coA dehydrogenase very long-chain (*ACADVL*), and acyl-coA dehydrogenase (*ACADM*). (C) PPARβ/δ responsive genes including pyruvate dehydrogenase kinase (*PDK*) and IL-1 (*ILK 1*). Values are LSM ± SEM scaled relative to control (n = 8 to 9 samples per treatment). Significant difference from control are indicated (*P < 0.05; **P < 0.01).

Mammary expression of *PPAR*-α, *PPAR*-β/δ, and *PPAR*-γ was not changed during CLA-induced or diet-induced MFD (Figure 3A). Of the PPAR-α target genes, peroxisomal acyl-coenzyme A oxidase (*ACOX*) and acyl-coenzyme A dehydrogenase (*ACADM*) were not changed but carnitine palmitoyltransferase 1 (*CPT1a*) and acyl-CoA dehydrogenase very long-chain (*ACADVL*) were increased during diet-induced MFD (*P* < 0.05 and *P* < 0.001, respectively; Figure 3B). The PPAR-β/δ target gene pyruvate dehydrogenase kinase (*PDK*) the and IL-1 (*ILK 1*) were not altered by CLA or diet-induced MFD (Figure 3C).

### Interaction of CLA and PPAR-γ Agonist and Antagonist in MAC-T Cells

**Figure 4.**
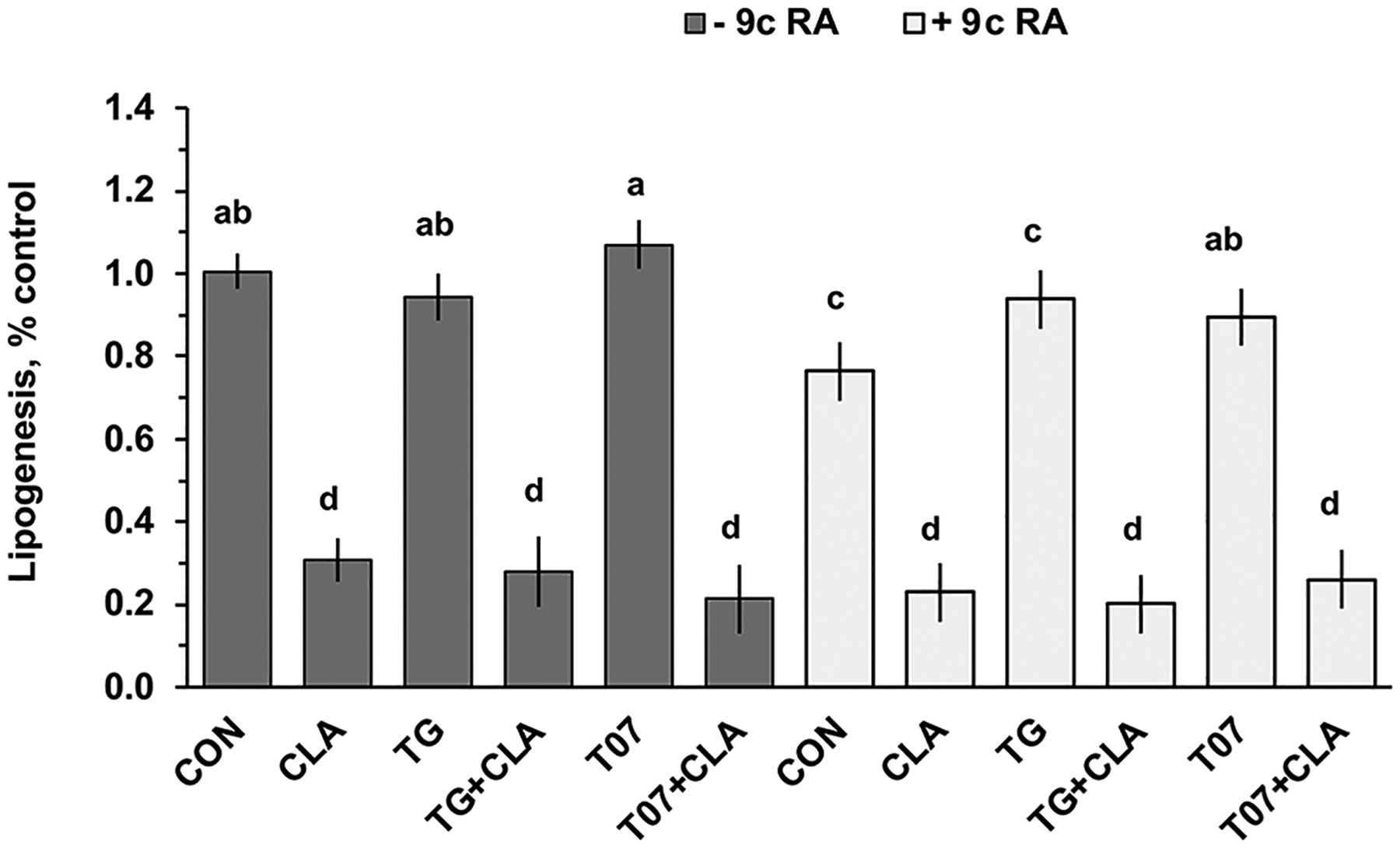
Effect of *trans-*10,*cis-*12 CLA and peroxisome proliferator-activated receptor gamma (PPAR-γ) agonist troglitazone (TG) and antagonist T0070907 (T07) on lipogenesis in a bovine mammary epithelial cell line (MAC-T). Lipogenesis was measured by incorporation of ^14^C acetate into lipids during the last 4 h of treatment and expressed relative to the control. Cells were treated for 24 h in basal medium with 75 μ*M trans-*10,*cis-*12 CLA, 5 μ*M* TG, 5 μ*M* T07, or combinations of these treatments in the presence or absence of 10 μ*M* of 9-*cis* retinoic acid (9c RA). Values represent LSM ± SEM (includes intra- and interexperimental run error). Means are scaled relative to control (control set to 100; n = 6 wells per treatment across 2 independent experiments). Means that do not share a letter (a–d) differ (*P* < 0.05).

Mammary cell culture was used to test the direct interaction of PPAR-γ agonist and antagonist. PPAR-γ agonist (TG) and antagonist (T07) did not affect lipogenesis in the absence of 9cRA, but in the presence of 9cRA, TG decreased lipogenesis by 15%, whereas T07 had no effect (Figure 4). *Trans*-10,*cis-*12 CLA reduced lipogenesis by ~70% (*P* < 0.05) in the presence or absence of RXR agonist 9cRA, and TG and T07 failed to overcome this effect. Similar responses were observed in media with and without supplementation with serum and insulin (data from serum-free shown).

**Figure 5.**
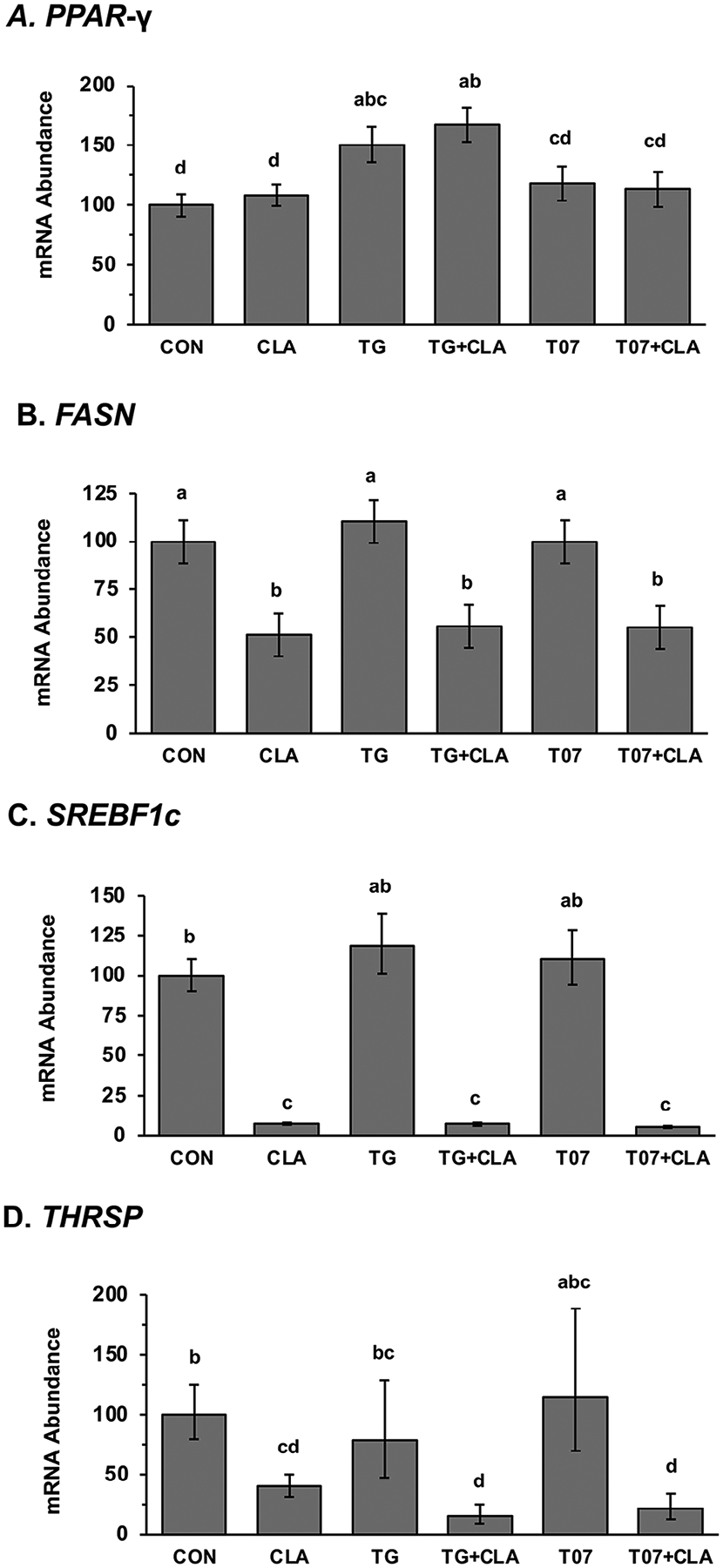
Effect of *trans-*10,*cis-*12 CLA and peroxisome proliferator-activated receptor gamma (PPAR-γ) agonist troglitazone (TG) and antagonist T0070907 (T07) on gene expression in a bovine mammary epithelial cell line (MAC-T). Cells were treated for 24 h in basal media with 75 μ*M* CLA, 5 μ*M* of the agonist or antagoinist, or combinations of these treatments. (A) Expression of *PPAR-*γ; (B) expression of fatty acid synthase (*FASN*); (C) expression of sterol response element binding factor 1c (*SREBF1c*); (D) expression of thyroid hormone responsive (*THRSP*). Values are LSM ± SEM scaled relative to control (n = 5 to 8 per treatment). Means that do not share a letter differ (a–d) differ (*P* < 0.05).

The PPAR-γ agonist (TG) increased the expression of *PPAR-*γ by ~30% (*P* < 0.05), whereas the antagonist had no effect (Figure 5). *Trans*-10,*cis-*12 CLA had no effect on *PPAR*-γ expression in the presence or absence of TG and T07.

The effect on lipogenic regulators and enzymes was also explored to determine the influence on transcriptional regulation. *Trans*-10,*cis-*12 CLA reduced expression of *SREBF1*, *THRSP*, and *FASN* and this repression was not altered by the presence of the TG and T07 (Figure 5). Specifically, CLA reduced *SREBF1* expression by 92.5%, *THRSP* by 60%, and *FASN* by 49%. Data from 9cRA and serum and insulin-free conditions are shown, but a similar response was observed in the presence of 9cRA and serum and insulin supplementation.

## DISCUSSION

Diet and CLA-induced MFD result in a decrease in mammary lipogenic capacity and a coordinated decrease in lipogenic enzymes (Harvatine et al., 2009). A reduction in mammary expression of *SREBF1*, a central lipogenesis regulator, has been consistently observed and is currently the leading established candidate mechanism. However, the direct link between bioactive CLA isomers and SREBF1 signaling has not been described. Some members of the nuclear receptor family also regulate lipid metabolism (Cariello et al., 2021). We have previously found little support for the functional role of the liver X receptors (LXR) in MFD (Harvatine et al., 2014). The PPAR isoforms have been suggested as candidates as they are known to regulate lipid metabolism and have ligand-binding pockets (Bionaz et al., 2013).

The PPAR family have critical roles in lipid metabolism in numerous tissues, where PPAR-α and PPAR-β/δ enhance FA oxidation, and PPAR-γ promotes FA transport and lipogenesis (Janani and Ranjitha Kumari, 2015; Bougarne et al., 2018; Szántó et al., 2021). The PPAR isoforms are predominantly post-transcriptionally activated through ligand activation and formation of the active heterodimer with 9-*cis* retinoic acid-activated RXR (Kersten et al., 2000). They are also associated with coactivators and corepressors, further complicating their signaling (Ricote and Glass, 2007; Viswakarma et al., 2010).

Tissue profiling was conducted first in the current work to determine the relative expression of the PPAR isoforms in the mammary gland relative to other lipogenic tissue. As expected, *PPAR*-α was highly expressed in liver, heart, skeletal muscle, and tissues with high lipid oxidation rates, and lowly expressed in the mammary tissue (Figure 1A). The ubiquitous expression of *PPAR*-β/δ agrees with other animal models (Oyekan, 2011; Figure 1B). Mammary expression of *PPAR*-α and *PPAR*-β/δ was higher in lactating compared with nonlactating tissue. However, the relevance of this observation is not clear as the bovine mammary gland has very low rates of long-chain FA oxidation in the fed state (Annison et al., 1967; Bickerstaffe et al., 1974), limiting a mechanism of increased oxidation.

Recent research has established strong roles for PPAR-γ in adipogenesis and as an adipocyte differentiation marker (Janani and Ranjitha Kumari, 2015; Szántó et al., 2021), although roles in insulin resistance and lipid synthesis in adipose tissue and lipid metabolism and inflammation in macrophages have also been demonstrated (Braissant et al., 1996; Huin et al., 2000; Meng et al., 2005). The high expression observed in adipose tissue in the current profiling supports a major role of PPAR-γ in adipose tissue metabolism in the cow, but the low expression in the mammary gland questions its importance for milk fat synthesis, even though it was slightly increased in lactating tissue. It is important to recognize that mammary biopsies result in a mixture of cell types, including macrophages, myoepithelial, and possibly adipocytes, in addition to the mammary epithelial cells (Cánovas et al., 2014). The increase in *PPAR*-γ during lactation may be due to an increase in macrophages and other white blood cells rather than an increase in the expression in mammary epithelial cells. It would be valuable for future work to quantify expression patterns of individual cell types.

It appears that activation of PPAR-γ does have some positive feedback on its transcription in bovine mammary epithelial cells based on the increase in expression during treatment with the agonist in the current experiment. However, the antagonist had no effect (Figure 5A). Similarly, Vargas-Bello-Pérez et al. (2019) observed increased *PPAR-γ* expression with rosiglitazone (**ROSI**) in goat mammary epithelial cells, and Sandri et al. (2018) observed that thiazolidinedione (**TZD**) increased mammary expression of *PPAR-γ* in dairy ewes. However, PPAR-β/δ agonist and antagonist did not impact its expression in bovine mammary epithelial cells (Lohakare et al., 2018). It is important to note that CLA did not disrupt agonist stimulation of PPAR-γ expression.

Effects of *trans-*10,*cis-*12 CLA on *PPAR*-γ expression was of interest given its ability to activate PPAR-*γ* in transactivation assay and ROSI stimulation of *PPAR-γ* expression. The current experiment does not support such stimulatory effects in agreement with Harvatine et al., (2018) who did not observe a change in mammary *PPAR*-γ during CLA-induced MFD. In addition, others have observed reduced rather than increased *PPAR*-γ expression. For example, Ma et al., (2022) observed a 35% decrease in mammary tissue *PPAR*-γ expression when feeding a high-concentrate diet that decreased milk fat by 12.7% and also stimulated expression of inflammatory factors. In mice, Kadegowda et al., (2013) reported that a very high dose of *cis-*9,*trans-*11 and *trans-*10,*cis-*12 CLA downregulated mammary PPAR-γ and lipogenic genes with concurrent induction of liver steatosis and inflammatory markers. The dose was ~180 mg/d versus 22.5 mg/d required for maximal inhibition, as reported by Harvatine and Bauman (2011). Foote et al. (2010) observed that high doses of CLA resulted in inflammation and steatosis, while lower doses had more specific effects on lipid synthesis. Diet-induced MFD has been associated with increasing inflammation and oxidative stress (Ma et al., 2022). Hence, the response of PPAR-γ at higher doses and during diet-induced MFD may be associated with these processes rather than lipid metabolism.

We assessed the expression of PPAR-α and PPAR-β/δ target genes because the PPAR have major regulation at the level of ligand activation. It is important to note that these genes are not solely regulated by each PPAR, but were selected because a PPAR isoform plays a major role in their regulation. Thus, modification of PPAR activity would be expected to drive changes in their expression, providing an endogenous reporter. Only PPAR-α target genes *CPT1a* and *ACADVL* exhibited increased expression following the LF-HO diet while remaining unaffected by CLA infusion. The lack of change during CLA-induced MFD indicates that these are not functionally essential to the decrease in milk fat synthesis and may be related to other factors, such as increased dietary fat or inclusion of fish oil in the diet-induced MFD treatment. Additionally, lipid oxidation is very low in lactating mammary tissue, as discussed above, and the magnitude of increased flux of FA through the oxidative pathway likely is very low. Lastly, there CLA equally decreased body fat in wild-type and PPAR-α knockout mice, indicating a PPAR-α-independent mechanism (Peters et al., 2001).

We conducted in vitro experiments using specific PPAR-γ chemical agonists and antagonists because ligand activation of PPAR-γ is the most mechanistically related to lipid synthesis. The CLA inhibition of lipogenesis was replicated in the MAC-T cells, and the failure of the PPAR-γ agonists (TG) or antagonists (T07) to overcome the effect of CLA indicates that the mechanism is independent of PPAR-γ modulation. Others have also explored PPAR-γ agonists in mammary cell culture. Vargas-Bello-Pérez et al. (2019) reported that ROSI, a PPAR-γ agonist, increased the transcription of *FASN*, *SCD1*, and *AGPAT6*, whereas the PPAR-γ antagonist GW9662 decreased the basal transcription of *ACACA*, *LPIN1*, *GPAM*, *DGAT1*, and *ACSL1*, and increased the transcription of *FASN*. Kadegowda et al. (2009) also reported that ROSI increased the transcription of *ACACA*, *FASN*, *SCD*, *LPIN1*, *AGPAT6*, *DGAT1*, *SREBF1*, *SREBF2*, and *INSIG1* by more than 40% without changes in lipid droplet formation compared with the control.

The interaction of PPAR-γ agonists and CLA has also been studied in vivo in lactating mice, sheep, and goats. Vyas et al. (2014) reported that 10 mg/kg daily injections of ROSI failed to overcome the effect of a high dose of CLA (fed at 1.5% of the diet) on milk fat concentration, pup growth, and lipogenic gene expression and protein abundance. Sandri et al. (2018) reported that an IV infusion of 4 mg/kg of TZD did not change milk fat concentration or overcome CLA inhibition of milk fat in dairy sheep. Jaaf et al. (2019) also observed that i.v. infusion of 8 mg/kg of BW per day of TZD did not change milk fat yield or lipogenic genes in mammary cells isolated from milk, although it did decrease plasma free FA and ketones, increased blood glucose, and modified adipose expression of some genes involved in insulin signaling.

Overall, the lack of lipogenic response to PPAR-γ agonist and failure to rescue the effect of CLA does not support a functional role in diet-induced MFD. It is important to note that antagonists have not been explored in vivo, but the failure of the agonist provides little support for future work in the area. There are also PPAR-γ ligand-dependent and independent repression mechanisms, but these mechanisms primarily function to reduce inflammatory and immune responses discussed below (Janani and Ranjitha Kumari, 2015).

Building on established suppression of key transcription factors, including *SREBF-1* and *THRSP*, and lipogenic enzymes, such as *FASN*, during MFD, we explored the potential involvement of PPAR-γ in these regulatory pathways. There was no interaction of the PPAR-γ agonists and antagonists on CLA downregulation of *SREBF1c*, *FASN*, and *THRSP* (Figure 4), indicating that the effects of CLA on these occur through mechanisms independent of direct PPAR-γ modulation.

We also tested the interaction with the RXR agonist to determine if RXR activation was interacting with PPAR-γ activity. The lack of interaction with 9c-RA in the current experiment also indicates independence from RXR activity. It is worth noting that many studies investigating PPAR-dependent mechanisms do not include an RXR activator, which may limit the response to PPAR activators in these systems.

The PPAR family has effects well beyond lipid metabolism, including regulating genes involved in cell proliferation, apoptosis, and inflammation (Bougarne et al., 2018; Montaigne et al., 2021; Murru et al., 2021). For example, ligand activation of PPAR-γ is well recognized as an important modulator of inflammatory responses, influencing cytokine, chemokine, and survival factor transcription, and counteracting pro-inflammatory transcription factors such as NF-κB, AP-1, and STAT (Janani and Ranjitha Kumari, 2015; Szántó et al., 2021). Indeed, a hematopoietic and endothelial cell-specific PPAR-γ knockout led to elevated levels of inflammatory molecules in milk (Wan et al., 2007).

The established role of PPAR-γ in enhancing adipose tissue lipid synthesis and deposition theoretically could affect milk fat by reducing plasma availability of FA and substrate for milk fat synthesis as suggested by Gutgesell et al. (2009), who investigated CLA in lactating rats. However, the predominant mechanism of MFD appears to be inhibition of mammary capacity for lipid synthesis rather than a substrate limitation (Harvatine et al., 2009).

## CONCLUSIONS

Overall, the experimental evidence available in the cow and mammary cell culture and the fundamental biology of PPAR isoforms fail to support a role for them as regulators of mammary lipogenesis or a functional part of the inhibition of lipogenesis by bioactive *trans*-FA. Although expression of the 3 PPAR isoforms is slightly increased in mammary tissue during lactation, its low expression relative to other tissues provides the first challenge to its importance and expression was not changed during MFD. Although PPAR-γ a agonist stimulated PPAR-γ expression in mammary epithelial cells it did not affect lipogenesis and the agonist and an antagonist failed to mitigate *trans*-10,*cis-*12 CLA inhibition of lipogenesis. The lack of effect of PPAR-γ agonists on milk fat in vivo in mice, goats, and sheep reported by others and the inability to rescue CLA-induced MFD similarly fails to support a PPAR-γ dependent mechanism. It is impossible to entirely rule out a PPAR-dependent effect of CLA without the availability of a cow with a mammary epithelial cell-specific deletion of each PPAR, but the lack of evidence from the available experiments should prioritize the investigation of non-PPAR candidate mechanisms.

## Nonstandard abbreviations used:

9cRA: *cis-*9 retinoic acid
AT: adipose tissue
FA: fatty acid
Lact: lactating mammary gland
LF-HO: low-forage, high-oil diet
Liv: liver
MAC-T: bovine mammary epithelial cells
MFD: milk fat depression
Musc: skeletal muscle
PPAR: peroxisome proliferator-activated receptor
PPRE: peroxisome proliferator regulatory elements
ROSI: rosiglitazone
RXR: retinoid X receptors
ROSI: rosiglitazone
T07: PPAR-γ antagonist T0070907
TG: troglitazone
TZD: thiazolidinedione
Uter: uterus
